# Development and validation of a survival prediction model in elder patients with community-acquired pneumonia: a MIMIC-population-based study

**DOI:** 10.1186/s12890-023-02314-w

**Published:** 2023-01-18

**Authors:** Na Li, Wenli Chu

**Affiliations:** 1 Department of Clinical Medicine, College of Medicine, Pingdingshan University, Pingdingshan, 467000 People’s Republic of China; 2 Department of Respiratory and Critical Care Medicine, The Second Affiliated Hospital of Xi’an Medical College, No. 167 Fangdong Street, Baqiao District, Xi’an, 710038 People’s Republic of China

**Keywords:** Survival prediction model, Elder patients, Community-acquired pneumonia, MIMIC

## Abstract

**Background:**

To develop a prediction model predicting in-hospital mortality of elder patients with community-acquired pneumonia (CAP) admitted to the intensive care unit (ICU).

**Methods:**

In this cohort study, data of 619 patients with CAP aged ≥ 65 years were obtained from the Medical Information Mart for Intensive Care III (MIMIC III) 2001–2012 database. To establish the robustness of predictor variables, the sample dataset was randomly partitioned into a training set group and a testing set group (ratio: 6.5:3.5). The predictive factors were evaluated using multivariable logistic regression, and then a prediction model was constructed. The prediction model was compared with the widely used assessments: Sequential Organ Failure Assessment (SOFA), Pneumonia Severity Index (PSI), systolic blood pressure, oxygenation, age and respiratory rate (SOAR), CURB-65 scores using positive predictive value (PPV), negative predictive value (NPV), accuracy (ACC), area under the curve (AUC) and 95% confidence interval (CI). The decision curve analysis (DCA) was used to assess the net benefit of the prediction model. Subgroup analysis based on the pathogen was developed.

**Results:**

Among 402 patients in the training set, 90 (24.63%) elderly CAP patients suffered from 30-day in-hospital mortality, with the median follow-up being 8 days. Hemoglobin/platelets ratio, age, respiratory rate, international normalized ratio, ventilation use, vasopressor use, red cell distribution width/blood urea nitrogen ratio, and Glasgow coma scales were identified as the predictive factors that affect the 30-day in-hospital mortality. The AUC values of the prediction model, the SOFA, SOAR, PSI and CURB-65 scores, were 0.751 (95% CI 0.749–0.752), 0.672 (95% CI 0.670–0.674), 0.607 (95% CI 0.605–0.609), 0.538 (95% CI 0.536–0.540), and 0.645 (95% CI 0.643–0.646), respectively. DCA result demonstrated that the prediction model could provide greater clinical net benefits to CAP patients admitted to the ICU. Concerning the pathogen, the prediction model also reported better predictive performance.

**Conclusion:**

Our prediction model could predict the 30-day hospital mortality in elder patients with CAP and guide clinicians to identify the high-risk population.

## Introduction

Community-acquired pneumonia (CAP), defined as pneumonia acquired outside the hospital, is one of the most common infectious diseases in clinical practice [1, 2]. The incidence of pneumonia increases with age, with a 10 times higher hospitalization rate in patients aged 65 years and older (about 2000 per 100,000 per year) than in the younger population [3, 4]. CAP remains a common cause of intensive care unit (ICU) admissions and in-hospital mortality in the elderly [5, 6]. Approximately 75% of CAP patients require hospitalization, up to 10% of them need to be admitted to the ICU, and the in-hospital mortality from 4 to 20.9% in these patients, which poses a huge burden on families and society [7, 8]. Therefore, early identification of elderly CAP patients with high in-hospital mortality is crucial to timely and effective intervention for prognosis improvement.

Several pneumonia severity scores include the Sequential Organ Failure Assessment (SOFA), Pneumonia Severity Index (PSI), CURB-65, and systolic blood pressure, oxygenation, age and respiratory rate (SOAR) have been developed and used to predict outcomes in patients with CAP [9–11]. These scores are useful in the management of patient risk stratification, but there is still a lack of accurate assessment with regard to patient mortality [12]. Moreover, a common limitation of the above score systems is that a lot of variables cannot be obtained within the first 24 h after admission [13]. In addition to the scoring methods, in recent years, many blood biomarkers have been shown to play crucial roles in the early diagnosis and prognosis of pneumonia, including CAP [14, 15]. Serum albumin (ALB) level was reported to be associated with in-hospital mortality in patients with CAP [12]. The value of the red cell distribution width (RDW) has also been found in predicting the prognosis in critically ill patients [15]. A study demonstrated that the ALB-RDW score is the independent factor of 90-day mortality in patients with severe CAP [16]. Another study indicated neutrophil to lymphocyte (NLR) ratio was a promising candidate predictor of unfavorable outcomes in CAP patients [17]. However, there is a lack of research establishing prediction models to predict in-hospital mortality in CAP patients based on these biomarkers. Therefore, it is necessary to incorporate these biomarkers and develop new prediction models to achieve early assessment of in-hospital mortality risk in elderly CAP patients and guide clinical decision-making.

Herein, this study aimed to conduct a prediction model to predict the in-hospital mortality in elderly CAP patients and to compare the predictive value of the prediction model with SOFA, PSI, SOAR, and CURB-65 scoring system. We developed a simpler prediction model that may be beneficial to the decrease of in-hospital mortality in elder patients with CAP.

## Methods

### Study design and population

This study was a retrospective cohort study, and all data were obtained from the Medical Information Mart for Intensive Care III (MIMIC-III) 2001–2012. The MIMIC-III is a large, single-center, freely available database, which contained the comprehensive and high-quality medical records of 50,000 patients admitted to ICU at the Beth Israel Deaconess Medical Center between 2001 and 2012 [18]. Study inclusion criteria were (1) aged ≥ 65 years old; (2) population diagnosed with CAP at the time of admission to ICU. The exclusion criteria of this study were as follows: (1) the number of predictive factors that were not recorded during the first 24 h of the ICU stay exceeds 30% of the total number of predictive factors. Since the clinical data in this study were collected from a publicly available database, there were no local or state ethical issues.

### Data collection

All the patients were inquired the clinical data, including (1) baseline characteristics: age (years), gender, marital status, ethnicity; (2) vital signs: heart rate (times/min), respiratory rate (breaths/min), temperature (°C), systolic blood pressure (SBP, mmHg), diastolic blood pressure (DBP, mmHg), mean arterial pressure (MAP, mmHg); (3) comorbidities: liver cirrhosis, congestive heart-failure (CHF), renal failure, chronic obstructive pulmonary disease (COPD), septic shock, effusion, emphysema, lung cancer, heart disease, diabetes mellitus (DM), respiratory failure, atrial fibrillation (AF), hyperlipidemia, malignant cancer; (4) scoring systems: SOFA score, Simplified Acute Physiology Score (SAPSII), SOAR score, PSI, CURB-65 score, Glasgow coma scales (GCS), International normalized ratio (INR), Elixhauser comorbidity score; (5) laboratory parameters: red blood cell (RBC, m/uL), white blood cells (WBC, K/uL), mean corpuscular volume (MCV, μm^3^), blood urea nitrogen (BUN), sodium (mEq/L), haematocrit, potassium (mEq/L), phosphate (mg/dL), calcium (mg/dL), magnesium (mg/dL), lactate (mmol/L), creatine kinase (IU/L), arterial pH, oxygen saturation (SpO2, %), partial carbon dioxide pressure (PCO2), partial oxygen pressure (PO2), fraction of inspired oxygen (FiO2); (6) pathogen: *Streptococcus pneumoniae*, *Klebsiella pneumoniae*, *Legionella pneumophila*, other *Streptococcu*s, *Staphylococcus*, *Ecoli*, *Candida*, *Acinetobacter*, *Clostridium*, *Citrobacter*, *Enterococcus*, *Pneumocystis pneumonia* (PCP), other bacteria, virus, fungus, yeast; (7) treatments: invasive ventilation, ventilation, vasopressor; (8) inflammatory biomarker: NLR, platelet–lymphocyte ratio (PLR), prognostic nutritional index (PNI), anion gap, hemoglobin/platelets ratio (HPR), RDW/BUN ratio, absolute neutrophil count/ (white blood cell count-neutrophil count) (dNLR), BUN/ALB ratio, platelet count*(lymphocytes/neutrophil) (SII). Blood tests, pathogen detections, and scoring systems were performed on the first day after admission.

### Variable definitions and outcome

CAP was defined as evidence of a pulmonary infiltrate on the chest radiograph and symptoms of lower respiratory infection, including cough, dyspnea, fever, and/or pleuritic chest pain, which were not acquired in a hospital or a nursing home.

PNI referred to 10 × ALB (g/dL) + 0.005 × lymphocytes count. SOFA score uses SBP ≤ 100 mmHg, respiratory rate ≥ 22/min, and altered cognitive state to identify high risk patients. The SOAR score identifies severe CAP using the following criteria (definitions of variables for data extraction are also listed below): SBP < 90 mmHg, PaO2/FiO2 ratio < 250, age ≥ 65, and respiratory rate ≥ 30 breaths/min. The CURB-65 index identifies high risk patients using the following criteria (definitions of variables for data extraction are also listed below): confusion, BUN ≥ 20 mg/dl, respiratory rate ≥ 30 breaths/min, SBP < 90 mmHg or DBP ≤ 60 mmHg, and age ≥ 65.

The study outcome was the 30-day in-hospital mortality rate. When the patient died in the hospital, the follow-up period ended. The median follow-up was 8 days.

### Statistics analysis

The t-test was used to evaluate the normally distributed data which was presented as mean ± standard deviation (Mean ± SD). Mann Whitney U test was used to evaluate the non-normally distributed variables and data were presented as median and quartile M (Q_1_, Q_3_). Fisher's exact test was used to analyze the enumeration data and data were described as the number of cases and constituent ratio N (%). Multiple imputation was performed by “mice” R package for missing values. Sensitivity analysis was performed by comparing the data before and after imputation.

To establish the robustness of predictor variables, the sample dataset was randomly partitioned into the training set group and the testing set group (ratio: 6.5:3.5). *P* < 0.05 was considered statistically significant. To determine the predictive factors, variables achieving a significance level were selected for multivariable Logistic analysis and other variables that are significant in other studies were included. Then stepwise regression was performed to construct the final prediction model. The performance of the prediction model was evaluated by the Hosmer–Lemeshow (H–L) goodness-of-fit test, using positive predictive value (PPV), negative predictive value (NPV), accuracy (ACC), area under the curve (AUC), and 95% confidence interval (CI). To compare the performance of our prediction model with the SOFA, PSI, SOAR, and CURB-65, the DeLong test was applied. In addition, the decision curve analysis (DCA) was used to assess the net benefit of the prediction model and SOFA, PSI, SOAR, CURB-65. The total population was divided into subgroups to verify our prediction model.

Multiple imputation, DCA curve, and Logistic regression were performed using R Software (version 4.0.3; The R Project for Statistical Computing, TX, USA). ROC curves and prediction results were completed using Python 3.8. All remaining analyses were performed using SAS software (version 9.4; SAS Institute Inc., Cary, NC, USA).

## Results

### Characteristics of the included patients

**Fig. 1:**
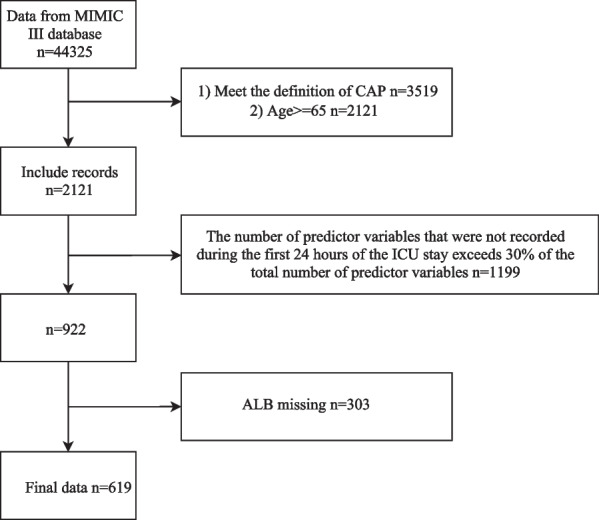
Flow chart of the participants selection

In total, 619 patients with CAP aged over 65 years were included in this study, with 217 patients in the testing set and 402 patients in the training set. The median follow-up was 8 days. The flow chart of the participants’ selection is shown in Fig. 1. The characteristics of the included patients between the testing set and the training set groups are described in Table 1. Among 402 patients in the training set, 90 (24.63%) elderly CAP patients suffered from 30-day in-hospital mortality; 41.41% (41 of 99) of the patients were male, while 58.59% (58 of 99) were women. The baseline and clinical characteristics between in-hospital mortality and non-in-hospital mortality are present in Table 2.

**Table 1:** Characteristics of the included patients between the testing set and the training set groups

Variables	Total (n = 619)	Testing set (n = 217)	Training set (n = 402)	Statistics	*P*
Age, years, Mean ± SD	78.44 ± 7.76	78.97 ± 7.47	78.16 ± 7.91	t = 1.24	0.217
Gender, n (%)				χ^2^ = 1.105	0.293
Female	296 (47.82)	110 (50.69)	186 (46.27)		
Male	323 (52.18)	107 (49.31)	216 (53.73)		
Marital status, n (%)				χ^2^ = 1.536	0.820
Divorced	34 (5.49)	15 (6.91)	19 (4.73)		
Married	298 (48.14)	105 (48.39)	193 (48.01)		
Separated	3 (0.48)	1 (0.46)	2 (0.50)		
Single	111 (17.93)	39 (17.97)	72 (17.91)		
Widowed	173 (27.95)	57 (26.27)	116 (28.86)		
Ethnicity, n (%)				χ^2^ = 7.042	0.134
Asian	29 (4.68)	12 (5.53)	17 (4.23)		
Black	62 (10.02)	19 (8.76)	43 (10.70)		
Hispanic	15 (2.42)	7 (3.23)	8 (1.99)		
Other	14 (2.26)	9 (4.15)	5 (1.24)		
White	499 (80.61)	170 (78.34)	329 (81.84)		
Respiratory rate, times/min, Mean ± SD	22.61 ± 6.94	22.41 ± 7.13	22.71 ± 6.84	t = − 0.52	0.606
Temperature, °C, Mean ± SD	36.80 ± 1.00	36.79 ± 0.99	36.80 ± 1.01	t = − 0.19	0.850
Heart rate, beats/min, Mean ± SD	95.65 ± 20.71	93.55 ± 20.03	96.79 ± 21.01	t = − 1.86	0.064
SBP, mmHg, Mean ± SD	121.75 ± 24.48	120.47 ± 26.35	122.45 ± 23.42	t = − 0.93	0.353
DBP, mmHg, Mean ± SD	61.67 ± 17.38	60.40 ± 17.73	62.36 ± 17.17	t = − 1.34	0.181
MAP, mmHg, M (Q_1_, Q_3_)	76.00 (67.00, 88.00)	74.00 (65.00, 88.00)	77.00 (67.67, 88.00)	Z = − 1.035	0.301
SpO2, % Mean ± SD	95.85 ± 6.33	95.69 ± 7.55	95.94 ± 5.57	t = − 0.43	0.666
WBC, K/Ul, M (Q_1_, Q_3_)	12.30 (8.70, 17.50)	12.90 (8.80,18.30)	12.00 (8.50,17.10)	Z = 1.510	0.131
RBC, m/uL, Mean ± SD	3.84 ± 0.72	3.79 ± 0.73	3.87 ± 0.71	t = − 1.17	0.243
Sodium, mEq/L, Mean ± SD	138.02 ± 6.19	137.95 ± 6.49	138.05 ± 6.03	t = − 0.19	0.851
Potassium, mEq/L, Mean ± SD	4.52 ± 0.89	4.54 ± 0.83	4.51 ± 0.93	t = 0.39	0.700
Phosphate, mg/dL, M (Q_1_, Q_3_)	3.50 (2.90, 4.30)	3.50 (3.00, 4.50)	3.40 (2.80, 4.20)	Z = 1.840	0.066
Calcium, mg/dL, Mean ± SD	8.46 ± 0.91	8.53 ± 0.83	8.42 ± 0.95	t = 1.46	0.144
pH, Mean ± SD	7.36 ± 0.11	7.36 ± 0.11	7.37 ± 0.11	t = − 1.07	0.286
Lactate, mmol/L, M (Q_1_, Q_3_)	2.00 (1.50, 2.90)	1.90 (1.40, 2.90)	2.00 (1.50, 2.90)	Z = − 0.634	0.526
INR, M (Q_1_, Q_3_)	1.20 (1.10, 1.60)	1.20 (1.10, 1.60)	1.30 (1.10, 1.60)	Z = − 0.195	0.845
MCV, μm^3^, Mean ± SD	91.10 ± 7.26	91.26 ± 7.64	91.01 ± 7.05	t = 0.41	0.680
magnesium, mg/dL, Mean ± SD	1.94 ± 0.39	1.96 ± 0.42	1.93 ± 0.37	t = 1.09	0.274
BUN, mg/dL, M (Q_1_, Q_3_)	29.00 (20.00, 43.00)	29.00 (20.00, 46.00)	29.00 (20.00, 43.00)	Z = 0.464	0.643
Creatine kinase, IU/L, M (Q_1_, Q_3_)	76.00 (38.00, 161.00)	63.00 (34.00, 137.00)	80.50 (40.00, 181.00)	Z = − 2.829	0.005
Hematocrit, %, Mean ± SD	34.79 ± 6.13	34.40 ± 6.12	35.00 ± 6.14	t = − 1.16	0.244
PO2, M (Q_1_, Q_3_)	84.00 (63.00, 133.00)	81.00 (62.00, 135.00)	85.50 (63.00, 129.00)	Z = − 0.492	0.623
Hemoglobin, g/dL, Mean ± SD	11.45 ± 2.07	11.30 ± 2.05	11.54 ± 2.08	t = − 1.38	0.169
PCO2, M (Q_1_, Q_3_)	42.00 (35.00, 52.00)	43.00 (35.00, 54.00)	42.00 (34.00, 51.00)	Z = 0.699	0.485
RDW, %, Mean ± SD	15.47 ± 2.19	15.55 ± 2.24	15.43 ± 2.16	t = 0.64	0.520
COPD, n (%)				χ^2^ = 0.000	0.987
No	479 (77.38)	168 (77.42)	311 (77.36)		
Yes	140 (22.62)	49 (22.58)	91 (22.64)		
Lung cancer, n (%)				χ^2^ = 3.392	0.066
No	592 (95.64)	212 (97.70)	380 (94.53)		
Yes	27 (4.36)	5 (2.30)	22 (5.47)		
AF, n (%)				χ^2^ = 0.395	0.530
No	350 (56.54)	119 (54.84)	231 (57.46)		
Yes	269 (43.46)	98 (45.16)	171 (42.54)		
Liver cirrhosis, n (%)				χ^2^ = 0.910	0.340
No	604 (97.58)	210 (96.77)	394 (98.01)		
Yes	15 (2.42)	7 (3.23)	8 (1.99)		
CHF, n (%)				χ^2^ = 0.149	0.700
No	293 (47.33)	105 (48.39)	188 (46.77)		
Yes	326 (52.67)	112 (51.61)	214 (53.23)		
Heart disease, n (%)				χ^2^ = 2.605	0.107
No	514 (83.04)	173 (79.72)	341 (84.83)		
Yes	105 (16.96)	44 (20.28)	61 (15.17)		
DM, n (%)				χ^2^ = 0.001	0.973
No	474 (76.58)	166 (76.50)	308 (76.62)		
Yes	145 (23.42)	51 (23.50)	94 (23.38)		
Respiratory failure, n (%)				χ^2^ = 0.801	0.371
No	243 (39.26)	80 (36.87)	163 (40.55)		
Yes	376 (60.74)	137 (63.13)	239 (59.45)		
Hyperlipidemia, n (%)				χ^2^ = 0.018	0.893
No	393 (63.49)	137 (63.13)	256 (63.68)		
Yes	226 (36.51)	80 (36.87)	146 (36.32)		
Renal failure, n (%)				χ^2^ = 0.110	0.740
No	268 (43.30)	92 (42.40)	176 (43.78)		
Yes	351 (56.70)	125 (57.60)	226 (56.22)		
Malignant cancer, n (%)				χ^2^ = 0.009	0.926
No	395 (63.81)	139 (64.06)	256 (63.68)		
Yes	224 (36.19)	78 (35.94)	146 (36.32)		
SAPSII, Mean ± SD	44.65 ± 13.24	44.78 ± 13.09	44.58 ± 13.34	t = 0.18	0.859
SOFA, M (Q_1_, Q_3_)	5.00 (3.00, 7.00)	5.00 (3.00, 7.00)	5.00 (3.00,7.00)	Z = 1.165	0.244
Septic shock, n (%)				χ^2^ = 0.492	0.483
No	500 (80.78)	172 (79.26)	328 (81.59)		
Yes	119 (19.22)	45 (20.74)	74 (18.41)		
Ventilation, n (%)				χ^2^ = 0.153	0.696
No	269 (43.46)	92 (42.40)	177 (44.03)		
Yes	350 (56.54)	125 (57.60)	225 (55.97)		
Vesopressor, n (%)				χ^2^ = 0.889	0.346
No	583 (94.18)	207 (95.39)	376 (93.53)		
Yes	36 (5.82)	10 (4.61)	26 (6.47)		
*Streptococcus pneumoniae*, n (%)				χ^2^ = 1.083	0.298
No	597 (96.45)	207 (95.39)	390 (97.01)		
Yes	22 (3.55)	10 (4.61)	12 (2.99)		
*Klebsiella pneumoniae*, n (%)				χ^2^ = 0.019	0.891
No	583 (94.18)	204 (94.01)	379 (94.28)		
Yes	36 (5.82)	13 (5.99)	23 (5.72)		
Virus, n (%)				χ^2^ = 1.020	0.312
No	602 (97.25)	213 (98.16)	389 (96.77)		
Yes	17 (2.75)	4 (1.84)	13 (3.23)		
Emphysema, n (%)				χ^2^ = 0.219	0.640
No	593 (95.80)	209 (96.31)	384 (95.52)		
Yes	26 (4.20)	8 (3.69)	18 (4.48)		
Pneumothorax, n (%)				χ^2^ = 0.008	0.927
No	608 (98.22)	213 (98.16)	395 (98.26)		
Yes	11 (1.78)	4 (1.84)	7 (1.74)		
Effusion, n (%)				χ^2^ = 0.350	0.554
No	544 (87.88)	193 (88.94)	351 (87.31)		
Yes	75 (12.12)	24 (11.06)	51 (12.69)		
Yeast, n (%)				χ^2^ = 0.479	0.489
No	414 (66.88)	149 (68.66)	265 (65.92)		
Yes	205 (33.12)	68 (31.34)	137 (34.08)		
Staphylococcus, n (%)				χ^2^ = 1.446	0.229
No	432 (69.79)	158 (72.81)	274 (68.16)		
Yes	187 (30.21)	59 (27.19)	128 (31.84)		
EColi, n (%)				χ^2^ = 7.523	0.006
No	583 (94.18)	212 (97.70)	371 (92.29)		
Yes	36 (5.82)	5 (2.30)	31 (7.71)		
Other bacteria, n (%)				χ^2^ = 0.017	0.895
No	447 (72.21)	156 (71.89)	291 (72.39)		
Yes	172 (27.79)	61 (28.11)	111 (27.61)		
Another *streptococcus*, n (%)				χ^2^ = 0.831	0.362
No	586 (94.67)	203 (93.55)	383 (95.27)		
Yes	33 (5.33)	14 (6.45)	19 (4.73)		
*Candida*, n (%)				χ^2^ = 1.499	0.221
No	587 (94.83)	209 (96.31)	378 (94.03)		
Yes	32 (5.17)	8 (3.69)	24 (5.97)		
*Acinetobacter*, n (%)				–	1.000
No	613 (99.03)	215 (99.08)	398 (99.00)		
Yes	6 (0.97)	2 (0.92)	4 (1.00)		
*Clostridium*, n (%)				χ^2^ = 0.744	0.388
No	596 (96.28)	207 (95.39)	389 (96.77)		
Yes	23 (3.72)	10 (4.61)	13 (3.23)		
*Citrobacter*, n (%)				–	1.000
No	617 (99.68)	216 (99.54)	401 (99.75)		
Yes	2 (0.32)	1 (0.46)	1 (0.25)		
*Enterococcus*, n (%)				χ^2^ = 0.088	0.767
No	562 (90.79)	196 (90.32)	366 (91.04)		
Yes	57 (9.21)	21 (9.68)	36 (8.96)		
PCP, n (%)				χ^2^ = 1.730	0.188
No	617 (99.68)	217 (100.00)	400 (99.50)		
Yes	2 (0.32)	0 (0.00)	2 (0.50)		
Fungus, n (%)				–	0.615
No	615 (99.35)	215 (99.08)	400 (99.50)		
Yes	4 (0.65)	2 (0.92)	2 (0.50)		
CURB-65, Mean ± SD	2.55 ± 0.81	2.60 ± 0.82	2.52 ± 0.80	t = 1.17	0.244
PSI, Mean ± SD	136.63 ± 27.27	136.91 ± 28.73	136.48 ± 26.49	t = 0.19	0.850
NLR, M (Q_1_, Q_3_)	10.40 (5.99, 18.52)	10.71 (6.20, 17.40)	9.92 (5.97, 19.98)	Z = − 0.037	0.971
PLR, M (Q_1_, Q_3_)	2.78 (1.70, 4.61)	2.90 (1.77, 4.30)	2.75 (1.69, 4.74)	Z = 0.112	0.911
SOAR, M (Q_1_, Q_3_)	2.00 (1.00, 2.00)	2.00 (1.00, 2.00)	2.00 (1.00, 2.00)	Z = − 0.671	0.502
HPR, M (Q_1_, Q_3_)	0.04 (0.03, 0.06)	0.04 (0.03, 0.06)	0.05 (0.03, 0.06)	Z = − 2.377	0.017
Elixhauser comorbidity score, M (Q_1_, Q_3_)	20.00 (12.00, 29.00)	21.00 (14.00, 29.00)	20.00 (12.00, 29.00)	Z = 0.767	0.443
PNI, Mean ± SD	31.63 ± 8.01	31.58 ± 6.04	31.65 ± 8.90	t = − 0.13	0.899
RDW/BUN ratio, Mean ± SD	5.29 ± 1.54	5.25 ± 1.45	5.32 ± 1.59	t = − 0.50	0.619
dNLR, Mean ± SD	− 1.01 ± 0.07	− 1.01 ± 0.01	− 1.01 ± 0.09	t = − 0.15	0.885
BUN/ALB ratio, M (Q_1_, Q_3_)	9.71 (6.28, 14.44)	10.00 (6.32, 14.62)	9.63 (6.21, 14.40)	Z = 0.392	0.695
ALB, Mean ± SD	3.07 ± 0.62	3.09 ± 0.60	3.06 ± 0.64	t = 0.61	0.545
Anion gap, Mean ± SD	63.03 ± 9.69	63.22 ± 10.35	62.93 ± 9.32	t = 0.36	0.721
ICU LOS, M (Q_1_, Q_3_)	3.20 (1.73, 7.58)	3.13 (1.77, 7.58)	3.25 (1.72, 7.43)	Z = − 0.409	0.682
In hospital mortality, n (%)				χ^2^ = 0.104	0.747
No	464 (74.96)	161 (74.19)	303 (75.37)		
Yes	155 (25.04)	56 (25.81)	99 (24.63)		

ICU, intensive care unit; LOS, length of stay; SBP, systolic blood pressure; DBP, diastolic blood pressure; MAP, mean arterial pressure; SpO2, oxygen saturation; WBC, white blood cells; RBC, red blood cell; INR, International normalized ratio; MCV, mean corpuscular volume; BUN, blood urea nitrogen; PO2, partial oxygen pressure; PCO2, partial carbon dioxide pressure; COPD, chronic obstructive pulmonary disease; AF, atrial fibrillation; CHF, congestive heart-failure; DM, diabetes mellitus; SOFA, Sequential Organ Failure Assessment; SAPSII, Simplified Acute Physiology Score; PCP, Pneumocystis pneumonia; PSI, Pneumonia Severity Index; NLR, neutrophil to lymphocyte; PLR, platelet–lymphocyte ratio; SOAR, systolic blood pressure, oxygenation, age and respiratory rate; HPR, hemoglobin/platelets ratio; PNI, prognostic nutritional index; RDW, red cell distribution width; dNLR, absolute neutrophil count/(white blood cell count-neutrophil count); ALB, albumin

**Table 2:** Characteristics between in-hospital mortality and non-in-hospital mortality among elderly CAP patients

Variables	Total (n = 402)	In-hospital mortality		Statistics	*P*
		No (n = 303)	Yes (n = 99)		
Age, years, Mean ± SD	78.16 ± 7.91	77.94 ± 7.92	78.82 ± 7.87	t = − 0.96	0.336
Gender, n (%)				χ^2^ = 1.245	0.264
Female	186 (46.27)	145 (47.85)	41 (41.41)		
Male	216 (53.73)	158 (52.15)	58 (58.59)		
Marital status, n (%)				Fisher	0.497
Divorced	19 (4.73)	17 (5.61)	2 (2.02)		
Married	193 (48.01)	143 (47.19)	50 (50.51)		
Separate	2 (0.50)	1 (0.33)	1 (1.01)		
Single	72 (17.91)	54 (17.82)	18 (18.18)		
Widowed	116 (28.86)	88 (29.04)	28 (28.28)		
Ethnicity, n (%)				χ^2^ = 4.822	0.306
Asian	17 (4.23)	12 (3.96)	5 (5.05)		
Black	43 (10.70)	32 (10.56)	11 (11.11)		
Hispanic	8 (1.99)	8 (2.64)	0 (0.00)		
Other	5 (1.24)	4 (1.32)	1 (1.01)		
White	329 (81.84)	247 (81.52)	82 (82.83)		
Liver cirrhosis, n (%)				χ^2^ = 0.001	0.980
No	394 (98.01)	297 (98.02)	97 (97.98)		
Yes	8 (1.99)	6 (1.98)	2 (2.02)		
CHF, n (%)				χ^2^ = 1.750	0.186
No	188 (46.77)	136 (44.88)	52 (52.53)		
Yes	214 (53.23)	167 (55.12)	47 (47.47)		
Renal failure, n (%)				χ^2^ = 2.936	0.087
No	176 (43.78)	140 (46.20)	36 (36.36)		
Yes	226 (56.22)	163 (53.80)	63 (63.64)		
COPD, n (%)				χ^2^ = 2.240	0.134
No	311 (77.36)	229 (75.58)	82 (82.83)		
Yes	91 (22.64)	74 (24.42)	17 (17.17)		
Septic shock, n (%)				χ^2^ = 8.528	0.003
No	328 (81.59)	257 (84.82)	71 (71.72)		
Yes	74 (18.41)	46 (15.18)	28 (28.28)		
Effusion, n (%)				χ^2^ = 0.023	0.878
No	351 (87.31)	265 (87.46)	86 (86.87)		
Yes	51 (12.69)	38 (12.54)	13 (13.13)		
Emphysema, n (%)				χ^2^ = 0.698	0.403
No	384 (95.52)	288 (95.05)	96 (96.97)		
Yes	18 (4.48)	15 (4.95)	3 (3.03)		
Pneumothorax, n (%)				χ^2^ = 0.461	0.497
No	395 (98.26)	297 (98.02)	98 (98.99)		
Yes	7 (1.74)	6 (1.98)	1 (1.01)		
Lung cancer, n (%)				χ^2^ = 0.088	0.767
No	380 (94.53)	287 (94.72)	93 (93.94)		
Yes	22 (5.47)	16 (5.28)	6 (6.06)		
Heart disease, n (%)				χ^2^ = 0.426	0.514
No	341 (84.83)	255 (84.16)	86 (86.87)		
Yes	61 (15.17)	48 (15.84)	13 (13.13)		
DM, n (%)				χ^2^ = 3.823	0.051
No	308 (76.62)	225 (74.26)	83 (83.84)		
Yes	94 (23.38)	78 (25.74)	16 (16.16)		
Respiratory failure, n (%)				χ^2^ = 6.901	0.009
No	163 (40.55)	134 (44.22)	29 (29.29)		
Yes	239 (59.45)	169 (55.78)	70 (70.71)		
Hyperlipidemia, n (%)				χ^2^ = 2.803	0.094
No	256 (63.68)	186 (61.39)	70 (70.71)		
Yes	146 (36.32)	117 (38.61)	29 (29.29)		
Malignant cancer, n (%)				χ^2^ = 2.055	0.152
No	256 (63.68)	187 (61.72)	69 (69.70)		
Yes	146 (36.32)	116 (38.28)	30 (30.30)		
AF, n (%)				χ^2^ = 0.829	0.363
No	231 (57.46)	178 (58.75)	53 (53.54)		
Yes	171 (42.54)	125 (41.25)	46 (46.46)		
Heart rate, beats/min, Mean ± SD	96.79 ± 21.01	96.74 ± 20.98	96.92 ± 21.21	t = − 0.07	0.942
Respiratory rate, times/min, Mean ± SD	22.71 ± 6.84	22.28 ± 6.87	24.03 ± 6.61	t = − 2.22	0.027
Temperature, °C, Mean ± SD	36.80 ± 1.01	36.85 ± 1.02	36.65 ± 0.99	t = 1.74	0.083
SOFA, M (Q_1_, Q_3_)	5.00 (3.00, 7.00)	4.00 (2.00, 7.00)	7.00 (4.00, 9.00)	Z = 5.173	< 0.001
SAPSII, Mean ± SD	44.58 ± 13.34	41.98 ± 11.77	52.57 ± 14.70	t = − 6.52	< 0.001
SOAR, M (Q_1_, Q_3_)	2.00 (1.00, 2.00)	2.00 (1.00, 2.00)	2.00 (1.00, 2.00)	Z = 3.563	< 0.001
PSI, Mean ± SD	136.48 ± 26.49	135.61 ± 27.65	139.13 ± 22.51	t = − 1.27	0.205
CURB-65, Mean ± SD	2.52 ± 0.80	2.41 ± 0.78	2.85 ± 0.77	t = − 4.84	< 0.001
GCS, M (Q_1_, Q_3_)	14.00 (8.00, 15.00)	14.00 (9.00, 15.00)	10.00 (6.00, 15.00)	Z = − 3.637	< 0.001
INR, M (Q_1_, Q_3_)	1.30 (1.10, 1.60)	1.20 (1.10, 1.50)	1.40 (1.20, 1.80)	Z = 3.060	0.002
Elixhauser comorbidity score, M (Q_1_, Q_3_)	20.00 (12.00, 29.00)	20.00 (12.00, 28.00)	23.00 (11.00, 33.00)	Z = 1.640	0.101
*Streptococcus pneumoniae*, n (%)				χ^2^ = 0.459	0.498
No	390 (97.01)	293 (96.70)	97 (97.98)		
Yes	12 (2.99)	10 (3.30)	2 (2.02)		
*Klebsiella pneumoniae*, n (%)				χ^2^ = 1.356	0.244
No	379 (94.28)	288 (95.05)	91 (91.92)		
Yes	23 (5.72)	15 (4.95)	8 (8.08)		
*Legionella pneumophila*, n (%)				χ^2^ = 1.356	0.244
No	402 (100.00)	303 (100.00)	99 (100.00)		
Other *Streptococcus*, n (%)				χ^2^ = 0.142	0.706
No	383 (95.27)	288 (95.05)	95 (95.96)		
Yes	19 (4.73)	15 (4.95)	4 (4.04)		
Virus, n (%)				χ^2^ = 0.018	0.894
No	389 (96.77)	293 (96.70)	96 (96.97)		
Yes	13 (3.23)	10 (3.30)	3 (3.03)		
Other bacteria, n (%)				χ^2^ = 10.753	0.001
No	291 (72.39)	232 (76.57)	59 (59.60)		
Yes	111 (27.61)	71 (23.43)	40 (40.40)		
Yeast, n (%)				χ^2^ = 7.565	0.006
No	265 (65.92)	211 (69.64)	54 (54.55)		
Yes	137 (34.08)	92 (30.36)	45 (45.45)		
*Staphylococcus*, n (%)				χ^2^ = 0.135	0.713
No	274 (68.16)	208 (68.65)	66 (66.67)		
Yes	128 (31.84)	95 (31.35)	33 (33.33)		
EColi, n (%)				χ^2^ = 0.025	0.874
No	371 (92.29)	280 (92.41)	91 (91.92)		
Yes	31 (7.71)	23 (7.59)	8 (8.08)		
*Candida,* n (%)				χ^2^ = 0.198	0.656
No	378 (94.03)	284 (93.73)	94 (94.95)		
Yes	24 (5.97)	19 (6.27)	5 (5.05)		
*Acinetobacter*, n (%)				Fisher	1.000
No	398 (99.00)	300 (99.01)	98 (98.99)		
Yes	4 (1.00)	3 (0.99)	1 (1.01)		
*Clostridium*, n (%)				χ^2^ = 0.260	0.610
No	389 (96.77)	294 (97.03)	95 (95.96)		
Yes	13 (3.23)	9 (2.97)	4 (4.04)		
*Citrobacter*, n (%)				Fisher	1.000
No	401 (99.75)	302 (99.67)	99 (100.00)		
Yes	1 (0.25)	1 (0.33)	0 (0.00)		
*Enterococcus*, n (%)				χ^2^ = 0.003	0.957
No	366 (91.04)	276 (91.09)	90 (90.91)		
Yes	36 (8.96)	27 (8.91)	9 (9.09)		
PCP, n (%)				χ^2^ = 1.134	0.287
No	400 (99.50)	301 (99.34)	99 (100.00)		
Yes	2 (0.50)	2 (0.66)	0 (0.00)		
Fungus, n (%)				Fisher	0.432
No	400 (99.50)	302 (99.67)	98 (98.99)		
Yes	2 (0.50)	1 (0.33)	1 (1.01)		
MCV, Mean ± SD	91.01 ± 7.05	90.59 ± 7.06	92.28 ± 6.90	t = − 2.08	0.038
Hematocrit, %, Mean ± SD	35.00 ± 6.14	35.22 ± 6.37	34.34 ± 5.34	t = 1.36	0.176
RBC, m/uL, Mean ± SD	3.87 ± 0.71	3.91 ± 0.73	3.74 ± 0.62	t = 2.28	0.024
Potassium, mEq/L, Mean ± SD	4.51 ± 0.93	4.49 ± 0.91	4.59 ± 0.98	t = − 0.91	0.364
Phosphate, mg/dL, M (Q_1_, Q_3_)	3.40 (2.80,4.20)	3.30 (2.80,4.10)	3.70 (3.00,4.50)	Z = 2.480	0.013
Calcium, mg/dL, Mean ± SD	8.42 ± 0.95	8.43 ± 0.96	8.38 ± 0.93	t = 0.45	0.653
Magnesium, Mean ± SD	1.93 ± 0.37	1.93 ± 0.36	1.91 ± 0.40	t = 0.42	0.676
Lactate, mmol/L, M (Q_1_, Q_3_)	2.00 (1.50, 2.90)	1.90 (1.50, 2.80)	2.20 (1.50, 3.70)	Z = 1.886	0.059
Creatine kinase, IU/L, M (Q_1_, Q_3_)	80.50 (40.00, 181.00)	80.00 (39.00, 183.00)	82.00 (40.00, 167.00)	Z = 0.237	0.813
pH, Mean ± SD	7.37 ± 0.11	7.38 ± 0.10	7.33 ± 0.14	t = 2.64	0.009
SpO2, Mean ± SD	95.94 ± 5.57	95.90 ± 6.00	96.07 ± 4.01	t = − 0.32	0.749
PO2, M (Q_1_, Q_3_)	85.50 (63.00,129.00)	84.00 (63.00, 128.00)	89.00 (65.00, 136.00)	Z = 0.343	0.731
PCO2, M (Q_1_, Q_3_)	42.00 (34.00, 51.00)	42.00 (35.00, 51.00)	39.00 (33.00, 52.00)	Z = − 0.757	0.449
Fio2, M (Q_1_, Q_3_)	35.00 (1.00, 70.00)	35.00 (1.00, 60.00)	40.00 (1.00, 100.00)	Z = 1.280	0.200
Invasive ventilation, n (%)				χ^2^ = 23.105	< 0.001
No	218 (54.23)	185 (61.06)	33 (33.33)		
Yes	184 (45.77)	118 (38.94)	66 (66.67)		
Ventilation, n (%)				χ^2^ = 18.792	< 0.001
No	177 (44.03)	152 (50.17)	25 (25.25)		
Yes	225 (55.97)	151 (49.83)	74 (74.75)		
Vesopressor, n (%)				χ^2^ = 29.794	< 0.001
No	376 (93.53)	295 (97.36)	81 (81.82)		
Yes	26 (6.47)	8 (2.64)	18 (18.18)		
NLR, M (Q_1_, Q_3_)	9.92 (5.97, 19.98)	9.40 (6.04, 19.60)	11.20 (5.71, 21.28)	Z = 0.789	0.430
PLR, M (Q_1_, Q_3_)	2.75 (1.69, 4.74)	2.72 (1.68, 4.70)	3.03 (1.78, 4.91)	Z = 0.458	0.647
PNI, Mean ± SD	31.65 ± 8.90	32.33 ± 9.33	29.59 ± 7.12	t = 3.06	0.002
Anion gap, Mean ± SD	16.39 ± 3.86	16.33 ± 3.92	16.58 ± 3.69	t = − 0.55	0.583
HPR, M (Q_1_, Q_3_)	0.46 (0.32, 0.63)	0.46 (0.32, 0.63)	0.48 (0.35, 0.70)	Z = 1.571	0.116
RDW/BUN, Mean ± SD	5.32 ± 1.59	5.16 ± 1.52	5.79 ± 1.74	t = − 3.47	< 0.001
dNLR, Mean ± SD	− 1.01 ± 0.09	− 1.01 ± 0.06	− 1.02 ± 0.15	t = 0.26	0.793
BUN/ALB, M (Q_1_, Q_3_)	9.63 (6.21, 14.40)	9.05 (5.71, 14.00)	11.00 (7.35, 16.18)	Z = 2.891	0.004
SII, M (Q_1_, Q_3_)	24.74 (13.09, 44.68)	26.36 (13.61, 45.36)	21.64 (11.00, 42.32)	Z = − 1.723	0.085
LOS, M (Q_1_, Q_3_)	3.25 (1.72, 7.43)	3.06 (1.66, 7.01)	4.13 (1.95, 9.05)	Z = 1.632	0.103

CAP, community-acquired pneumonia; CHF, congestive heart-failure; COPD, chronic obstructive pulmonary disease; DM, diabetes mellitus; AF, atrial fibrillation; SOFA, sequential Organ Failure Assessment; SAPSII, Simplified Acute Physiology Score; SOAR, systolic blood pressure, oxygenation, age and respiratory rate; PSI, Pneumonia Severity Index; GCS, Glasgow coma scales; INR, International normalized ratio; MCV, mean corpuscular volume; RBC, red blood cell; SpO2, oxygen saturation; PCO2, partial carbon dioxide pressure; Fio2, fraction of inspired oxygen; NLR, neutrophil to lymphocyte; PLR, platelet–lymphocyte ratio; PNI, prognostic nutritional index; HPR, hemoglobin/platelets ratio; RDW, red cell distribution width; BUN, blood urea nitrogen; dNLR, absolute neutrophil count/ (white blood cell count-neutrophil count); SII, platelet count*lymphocytes/neutrophil; LOS, length of stay

### Identifications of the predictive factors for in-hospital mortality of elderly CAP patients

Factors achieving a significance level in the comparison of the in-hospital mortality and non-in-hospital mortality groups were septic shock (*P* = 0.003), respiratory failure (*P* = 0.009), respiratory rate (*P* = 0.027), SOFA score (*P* < 0.001), SAPSII score (*P* < 0.001), SOAR (*P* < 0.001), CURB-65 score (*P* < 0.001), GCS (*P* < 0.001), INR (*P* = 0.002), other bacteria (*P* = 0.001), yeast (*P* = 0.006), MCV (*P* = 0.038), RBC (*P* = 0.024), phosphate (*P* = 0.013), Ph (*P* = 0.009), invasive ventilation (*P* < 0.001), ventilation (*P* < 0.001), vasopressor (*P* < 0.001), PNI (*P* = 0.002), RDW/BUN ratio (*P* < 0.001), BUN/ALB ratio (*P* = 0.004) (Table 2). The above significant variables were included in multivariate logistic regression, and the significant variables in other studies were added to the multivariate model, including HPR, age, gender, ethnicity, anion gap, and Elixhauser comorbidity score. Finally, HPR [risk ratio (RR): 1.429, 95% CI 1.030–1.985, Pr > Chi-square: 0.033], age (RR: 1.017, 95% CI 0.984–1.05, Pr > Chi-square: 0.325), respiratory rate (RR: 1.049, 95% CI 1.012–1.087, Pr > Chi-square: 0.009), INR (RR: 1.363, 95% CI 1.077–1.724, Pr > Chi-square: 0.010), ventilation (RR: 1.974, 95% CI 1.084–3.592, Pr > Chi-square: 0.026), vesopressor (RR:4.201, 95% CI 1.643–10.743, Pr > Chi-square: 0.003), RDW/BUN (RR: 1.192, 95% CI 1.029–1.38, Pr > Chi-square: 0.019), GCS (RR: 0.919, 95% CI 0.861–0.982, Pr > Chi-square: 0.013) were the predictive factors for in-hospital mortality of elderly CAP patients. The predictive factors for in-hospital mortality of elderly CAP patients are shown in Table 3.

**Table 3:** The predictive factors and prediction model for in-hospital mortality of elderly CAP patients

Variables	Estimate	RR (95% CI)	Standard error	Wald statistics	Pr > Chi-square
HPR	0.357	1.429 (1.03–1.985)	0.167	4.553	0.033
Age	0.016	1.017 (0.984–1.050)	0.017	0.970	0.325
Respiratory rate	0.048	1.049 (1.012–1.087)	0.018	6.839	0.009
INR	0.309	1.363 (1.077–1.724)	0.120	6.639	0.010
Ventilation (Yes)	0.680	1.974 (1.084–3.592)	0.306	4.951	0.026
Vesopressor (Yes)	1.435	4.201 (1.643–10.743)	0.479	8.979	0.003
RDW/BUN	0.175	1.192 (1.029–1.380)	0.075	5.509	0.019
GCS	− 0.084	0.919 (0.861–0.982)	0.034	6.183	0.013

CAP, community-acquired pneumonia; HPR, hemoglobin/platelets ratio; RR: risk ratio; CI: confidence interval; INR: International normalized ratio; RDW, red cell distribution width; BUN, blood urea nitrogen; GCS, Glasgow coma scales

### Prediction model construction and evaluations

Based on the predictive factors, the prediction model for predicting in-hospital mortality of elderly CAP patients was constructed. Table 3 shows the detailed information of our prediction model. The final prediction model is.

y = − 4.83 + 0.36*HPR + 0.016age + 0.05*respiratory rate + 0.31*INR + 0.68*ventilation (yes = 1, no = 0) + 1.44*vasopressor (yes = 1, no = 0) + 0.176*RDW/BUN − 0.08GCS.

**Fig. 2:**
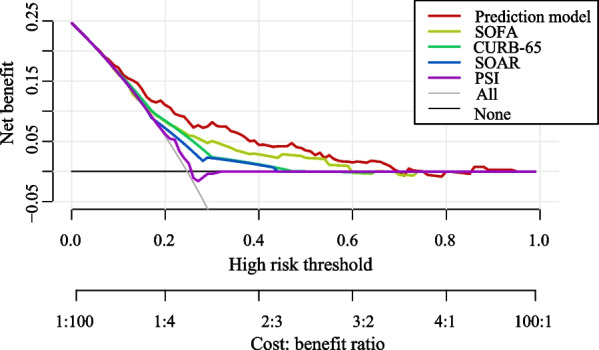
DCA result of the prediction model, SOFA, SOAR, PSI, and CURB-65 scores

The chi-square and *P* values of the H–L goodness of fit of our model in the training set and testing set were χ^2^ = 3.502, *P* = 0.899, χ^2^ = 7.196, *P* = 0.516, respectively, indicating that our model showed good goodness-of-fit. In addition, In the training set, the PPV, NPV, AUC, and ACC of the prediction model were 0.529 (95% CI 0.433–0.626), 0.850 (95% CI 0.810–0.890), 0.751 (95% CI 0.749–0.752), 0.769 (95% CI 0.727–0.810), respectively. The results of the prediction model evaluation are described in Table 4. The DCA results indicated that the prediction model had a good ability for predicting in-hospital mortality of elderly CAP patients. DCA result of the prediction model is shown in Fig. 2.

**Table 4:** Performances of the prediction model and scoring systems

Indicators	PPV (95% CI)	NPV (95% CI)	AUC (95% CI)	ACC (95% CI)
Training set				
Prediction model	0.529 (0.433–0.626)	0.850 (0.810–0.890)	0.751 (0.749–0.752)	0.769 (0.727–0.810)
SOFA	0.448 (0.348–0.547)	0.817 (0.774–0.860)	0.672 (0.670–0.674)*	0.729 (0.685–0.772)
SOAR	0.302 (0.242–0.362)	0.825 (0.769–0.881)	0.607 (0.605–0.609)*	0.532 (0.484–0.581)
PSI	0.273 (0.225–0.321)	0.875 (0.799–0.951)	0.538 (0.536–0.540)*	0.381 (0.333–0.428)
CURB-65	0.332 (0.267–0.396)	0.843(0.792–0.893)	0.645 (0.643–0.646)*	0.582 (0.534–0.630)
Testing set				
Prediction model	0.475 (0.350–0.601)	0.827 (0.768–0.886)	0.744 (0.742–0.747)	0.728 (0.669–0.787)
SOFA	0.356 (0.216–0.495)	0.767 (0.704–0.831)	0.634 (0.632–0.637)*	0.682 (0.620–0.744)
SOAR	0.360 (0.271–0.450)	0.849 (0.781–0.917)	0.667 (0.665–0.670)	0.599 (0.534–0.664)
PSI	0.301 (0.233–0.369)	0.927 (0.847–1.000)	0.621 (0.618–0.623)*	0.419 (0.354–0.485)
CURB	0.538 (0.347–0.730)	0.780 (0.721–0.839)	0.671 (0.668–0.673)*	0.751 (0.694–0.809)
External validation				
Prediction model	0.467 (0.418–0.516)	0.790 (0.764–0.817)	0.703 (0.672–0.734)	0.692 (0.666–0.717)
SOFA	0.448 (0.403–0.494)	0.801 (0.774–0.828)	0.686 (0.654–0.718)	0.675 (0.649–0.701)
SOAR	0.327 (0.294–0.361)*	0.766 (0.731–0.802)	0.564 (0.533–0.594)*	0.511 (0.484–0.539)*
PSI	0.379 (0.335–0.423)*	0.762 (0.734–0.791)	0.613 (0.580–0.646)*	0.626 (0.599–0.653)*
CURB-65	0.329 (0.295–0.362)*	0.769 (0.734–0.805)	0.570 (0.538–0.601)*	0.511 (0.484–0.539)*

PPV, positive predictive value; NPV, negative predictive value; AUC, area under the curve; ACC, accuracy; CI: confidence interval; SOFA, Sequential Organ Failure Assessment; SOAR, systolic blood pressure, oxygenation, age and respiratory rate; PSI, Pneumonia Severity Index; * is statistically significant compared with the prediction model

For the external validation of the prediction model, the result showed that the AUC our prediction model was 0.703, higher than SOFA (AUC: 0.686), SOAR (0.564), PSI (0.613), and CURB-65 (0.570).

### Comparison of the predictive performances between the prediction model and SOFA, SOAR, PSI, and CURB-65 scores

**Fig. 3:**
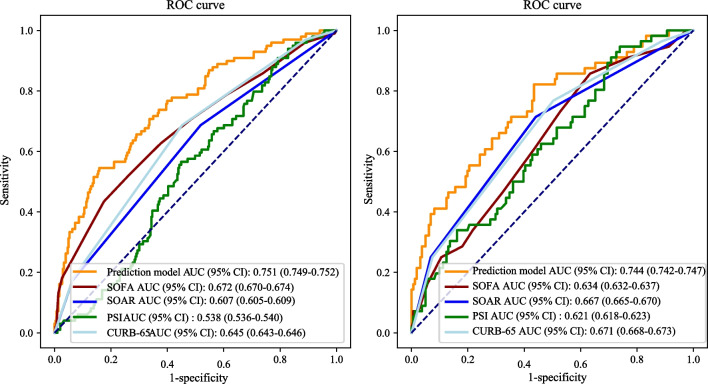
The AUC values of the prediction model, SOFA, SOAR, PSI, and CURB-65 scores in training set and testing set

We calculated the AUC value of the prediction model and four other scoring systems as shown in Fig. 3. The AUC value of the prediction model was 0.751 (95% CI 0.749–0.752), while those of the SOFA, SOAR, PSI and CURB-65 scores were 0.672 (95% CI 0.670–0.674), 0.607 (95% CI 0.605–0.609), 0.538 (95% CI 0.536–0.540), and 0.645 (95% CI 0.643–0.646), respectively. PPV, NPV, and ACC were also calculated to compare the predictive performance of the prediction model and SOFA, SOAR, PSI, and CURB-65 scores. Details of the performance are shown in Table 4.

### The predictive performances of the prediction model and SOFA, SOAR, PSI, and CURB-65 scores in the subgroup analysis based on the pathogen

Based on bacteria pathogen, the prediction model showed a higher AUC compared with SOFA, SOAR, PSI and CURB-65 scores, with AUC being 0.727 (95% CI 0.725–0.729), 0.632 (95% CI 0.630–0.634), 0.617(95% CI 0.616–0.619), 0.563(95% CI 0.561–0.565), 0.640 (95% CI 0.639–0.642), respectively. Concerning other pathogens, the prediction model also reported better predictive performance. The predictive performances of the prediction model and SOFA, SOAR, PSI, and CURB-65 scores based on the pathogen are shown in Table 5.

**Table 5:** The predictive performances of the prediction model in the subgroup analysis based on the pathogen

Indicators	PPV (95% CI)	NPV (95% CI)	AUC (95% CI)	ACC (95% CI)
Bacteria				
Prediction model	0.696 (0.563–0.829)	0.764 (0.720–0.809)	0.727 (0.725–0.729)	0.756 (0.714–0.799)
Other pathogen				
Prediction model	0.636 (0.352–0.921)	0.841 (0.792–0.890)	0.751 (0.748–0.753)	0.831 (0.782–0.880)

PPV, positive predictive value; NPV, negative predictive value; AUC, area under the curve; ACC, accuracy; CI: confidence interval

## Discussion

CAP is a global infectious disease that causes high morbidity and mortality. Accurate and timely identification of patients at high risk of mortality is one of the most important works of physicians. In this study, patients with CAP 30-day mortality rate was 24.63%, slightly higher than the previous related literature reports, this may be because of the large cases included in the ICU, and the age, basic diseases, or a minority of the critically ill patient into the hospital later on. We identified HPR, RDW/BUN ratio, age, respiratory rate, INR, GCS, ventilation use, and vasopressor use were independent predictive factors that were related to the hospital mortality of elderly CAP patients. Based on the predictive factors, our prediction model showed a better predictive performance with an AUC being 0.751. Our model also showed good goodness-of-fit. The prediction model also demonstrated better predictive performances than the SOFA, PSI, SOAR, and CURB-65 scoring systems.

The widespread application of scoring systems in clinical practice has brought great benefits to the management of CAP. However, these assessment scales do have limitations. PSI contains 20 variables and is very complex for prediction, these variables are usually not available at the initial visit, which may affect its promotion and implementation in daily practice. The CURB-65 score may not perform a suitable outcome in patients over the age of 70 due to its low sensitivity [19]. The SOFA score is unduly dependent on clinical therapeutic interventions which fail to be mastered easily [13]. SOAR might be more suitable for assessing disease severity, particularly in the elderly [19]. However, SOAR for in-hospital mortality needs further confirmation. Furthermore, our study showed that these scoring systems have only moderate AUC. Zhang et al. conducted a new prediction model for assessing the clinical outcomes of ICU patients with CAP [20]. However, the AUC of this model was 0.661. Another study with age, congestive heart failure, dementia, respiratory rate and BUN level being predictive factors to develop a prediction model to predict 1-year mortality after hospitalization for CAP. Nevertheless, the C-index was only. 0.76. In addition, the model only focuses on out-of-hospital mortality and only focuses on single biomarkers. The variables considered in our prediction model are more complete. Based on the predictive factors including HPR, RDW/BUN ratio, age, respiratory rate, INR, GCS, ventilation use, and vasopressor use, our prediction model was conducted. our prediction model demonstrated a higher AUC than the scoring systems, with an AUC being 0.751. Moreover, our prediction model was constructed based on the predictive factor that has the benefit of being easily calculated and not dependent on operator capacity to correctly gauge the level of confusion in a patient.

Our study demonstrated that RDW/BUN ratio and HPR were associated with in-hospital mortality of elderly CAP patients. The predictive value of the RDW/BUN ratio on the in-hospital mortality of elderly CAP patients may be due to the prognostic value of RDW and BUN. The RDW can be obtained immediately from blood routine reports, which has been found to be associated with mortality in patients with CAP [21, 22]. Ge et al. also confirmed that elevated RDW and WBC increased mortality in adult CAP patients [23]. Inflammation and oxidative stress caused by infection were thought to be the mechanisms of RDW and infectious diseases. Erythropoietin regulates myelogenesis, red cell maturation, and survival and was previously considered to be one of the major determinants of RDW [24]. Abnormal production of erythropoietin or the body's low response to erythropoietin will lead to a gradual increase in RDW value [25]. CAP is a typical infectious disease, during which Inflammation stimulates the release of inflammatory factors, damages the activity of erythropoietin, prevents the maturation of RBCs, leads to the production of ineffective RBCs, increases the heterogeneity of RBC size, and RDW value [26]. Increased RDW values have been reported to be correlated with inflammatory markers, indicating that C-reactive protein (CRP) and erythrocyte sedimentation rate (ESR) are high when RDW is high [27]. BUN is produced by the metabolism of protein and amino acids in the body, then hydrolyzed by the liver and excreted by the kidney with urine [28]. Due to this complex interplay of modulatory factors, BUN is generally used as a surrogate marker of systemic illness rather than a specific marker of renal dysfunction [29]. BUN ≥ 7 mmol was one of the CURB-65 scoring criteria for CAP [30]. In a retrospective study by Kang et al. in China that evaluated 4880 patients aged ≥ 65 with CAP, BUN was a prognostic factor for in-hospital mortality [31]. Uematsu et al. reported that elevated BUN had a significantly higher risk of 30-day mortality in CAP patients [32]. RDW/BUN ratio may be a simple and potentially useful prognostic factor of in-hospital mortality in elderly CAP patients. The HPR was calculated based on the hemoglobin and platelet counts. Patients with CAP often exhibit a declining hemoglobin concentration [33]. A study of patients with CAP found that hemoglobin levels < 10 g/dL were independently associated with 90-day mortality [34]. Abnormal platelet count has previously been related to different complications in patients with CAP admitted to ICU [35]. Rising platelet count throughout hospitalization has been found to be a powerful predictor of better survival, while declining platelet count predicts poor outcomes [36]. Tang et al. found that preoperative HPR can be taken into account as a factor predictive of oncological outcomes for stage 1 and grade 3 bladder cancer, particularly disease progression and mortality outcomes [37]. The HPR can be used as a veritable blood biomarker to predict the in-hospital mortality of elder patients with CAP.

Age, respiratory rate, ventilation use, vesopressor use, GCS could also predict the in-hospital mortality of elderly CAP patients in this study. A study evaluating the prognostic factors in hospitalized CAP identified age, respiratory rate, and mechanical ventilation as prognostic factors of in-hospitalized CAP patients [30]. Braunet al. found that variables associated with an increased risk of 90-day mortality included age ≥ 51 years [38]. A study by Baek et al. found that mechanical ventilation was associated with in-hospital mortality of pneumonia [39]. GCS is the most widely accepted tool for evaluating consciousness [40]. Wang et al. identified GCS as an independent predictor that was closely related to the hospital mortality of severe CAP [13].

Our predictive factors are routinely and rapidly measured in patients in a hospital setting; our prediction model therefore may be a useful early tool in predicting elderly CAP patients with a high risk of in-hospital mortality, who require rapid and timely decision making. There are several limitations to this study. First, considering the retrospective design, and the limitation of the sampling analysis, selection and sampling bias cannot be excluded. Second, this study only included hospitalized patients; therefore, it is difficult to generalize these findings to all CAP patients. Third, as no external validation was conducted in this study, the applicability of the prediction model in clinical practice requires further study. Therefore, the results should be interpreted cautiously when applied in other clinical settings. Further multicenter studies with populations of different geographic areas, a larger number of subjects, and above all, a prospective design are needed to corroborate the additive value of these markers to clinical prediction models to provide a safer and more effective assessment tool for clinicians.

## Conclusion

HPR, age, respiratory rate, INR, ventilation use, vasopressor use, RDW/BUN ratio, and GCS can be used as the factors to predict the in-hospital mortality of elderly CAP patients. The prediction model based on these predictive factors can help clinicians to make clinical decisions timely and early, and decrease the in-hospital mortality of elder patients with CAP admitted to the ICU.

## Data Availability

The datasets generated and/or analyzed during the current study are available in the MIMIC-III database, https://mimic.mit.edu/docs/gettingstarted/.

## Abbreviations

CAP: Community-acquired pneumonia
ICU: Intensive care unit
SOFA: Sequential Organ Failure Assessment
PSI: Pneumonia Severity Index
ALB: Serum albumin
MIMIC-III: Medical Information Mart for Intensive Care III
SBP: Systolic blood pressure
DBP: Diastolic blood pressure
MAP: Mean arterial pressure
CHF: Congestive heart-failure
COPD: Chronic obstructive pulmonary disease
DM: Diabetes mellitus; atrial fibrillation
SAPSII: Simplified Acute Physiology Score
GCS: Glasgow coma scales
INR: International normalized ratio
RBC: Red blood cell
WBC: White blood cell
MCV: Mean corpuscular volume
BUN: Blood urea nitrogen
SpO_2_: Oxygen saturation
PCO_2_: Partial carbon dioxide pressure
PO_2_: Partial oxygen pressure
FiO_2_: Fraction of inspired oxygen
PCP: Pneumocystis pneumonia
PLR: Platelet–lymphocyte ratio
PNI: Prognostic nutritional index
